# Case Report: A Novel Non-Canonical Splice Site Variant (c.1638+7T>C) in *TRPM6* Cause Primary Homagnesemia With Secondary Hocalcemia

**DOI:** 10.3389/fped.2022.834241

**Published:** 2022-05-25

**Authors:** Jiayu Song, Juan Lei, Jianxia Zhang, Aiqing Zhang, Weihua Gan, Bixia Zheng, Chunli Wang, Jing Gong

**Affiliations:** ^1^Department of Pediatric Nephrology, The Second Affiliated Hospital of Nanjing Medical University, Nanjing, China; ^2^Nanjing Key Laboratory of Pediatrics, Children's Hospital of Nanjing Medical University, Nanjing, China

**Keywords:** primary hypomagnesemia, secondary hypocalcemia, *TRPM6* gene, variant, abnormal mRNA splicing

## Abstract

**Objective:**

Primary hypomagnesemia with secondary hypocalcemia (HSH) is caused by loss-of-function mutations in the *TRPM6* gene encoding the epithelial magnesium channel. It is characterized by hypomagnesemia and secondary hypocalcemia associated with neurological symptoms. Here, we aimed to investigate the genetic defects of the *TRPM6* gene found in a girl from China.

**Methods:**

The genomic DNA of the proband and the parents was extracted for whole-exome sequencing. Sanger sequencing was further performed to validate the candidate variants. Subsequently, the *TRPM6* gene deletion was verified by quantitative PCR (qPCR) experiment. The effect of the variant on mRNA splicing was analyzed through a minigene splice assay and reverse transcription PCR (RT-PCR) *in vitro*.

**Results:**

The proband presented with the symptoms of generalized seizures, tetany, and muscle spasms, which were refractory to anticonvulsant treatment. Phenotypic data indicated that the patient had hypomagnesemia, poor parathyroid hormone response, and resultant hypocalcemia. The trio whole-exome sequencing identified that the proband carried compound heterozygous variants in the *TRPM6* gene, a paternally derived exon 6 deletion, and a maternally derived splicing variant (c.1638+7T>C) in exon 14. The minigene splice assay confirmed that the c.1638+7T>C variant resulted in exon 14 skipping, which caused the alteration of *TRPM6* mRNA splicing.

**Conclusion:**

Our results support that the compound heterozygous variants in *TRPM6* are responsible for HSH in this patient. A novel pathogenic splicing variant (c.1638+7T>C) in the intron 14 disturbs the normal *TRPM6* mRNA splicing, suggesting that the non-classical splice variant plays a critical role in HSH. This variant is essential for future effective genetic diagnosis.

## Introduction

Primary hypomagnesemia with secondary hypocalcemia (HSH; OMIM, #602014) is a rare autosomal recessive inherited metabolic disorder distinguished by profound hypomagnesemia, secondary hypocalcemia, and unreasonably low parathyroid hormone levels (1). HSH is caused by the loss of function of the epithelial magnesium channel protein encoded by the transient receptor potential cation channel subfamily M member 6 (*TRPM6*) gene (2). *TRPM6* is mapped to chromosome 9q22 and expressed in intestinal epithelial cells and kidney tubules, which predominantly mediates intestinal Mg^2+^ absorption and renal Mg^2+^ excretion (3). Hypocalcemia is caused due to a decrease in the release of parathyroid hormone (PTH) as a result of severe hypomagnesemia (4). Reportedly, pathogenic *TRPM6* mutation sites are associated with HSH disease (5, 6). According to the Human Genome Mutation Database (HGMD; http://www.hgmd.cf.ac.uk), only 77 mutations have been identified in the *TRPM6* gene, involving 37 missense mutations or non-sense mutations, 14 splice site mutations, 13 small deletions, three small insertions, one small indels, and nine gross deletions. In this study, we identify a novel splice site variant (c.1638+7T>C) which disturbed the normal mRNA splicing *in vitro* and exon 6 deletions, suggesting that compound heterozygous *TRPM6* gene variants play a significant role in the pathogenesis of HSH. Moreover, supplementation with calcium and magnesium can improve the clinical symptoms of HSH.

## Materials and Methods

### Case Description

The proband was a girl who was born full-term with normal growth and development with an uncomplicated perinatal period. Her mother and father were in good health were in good health. She developed a carpopedal spasm at birth and was referred to The Second Affiliated Hospital of Nanjing Medical University. She had negative imaging and electrophysiological findings in EEG. Her workups revealed that total serum calcium was 1.19 nmol/L (normal: 2.0–2.7 nmol/L), ion calcium was 0.64 nmol/L (normal: 1.1–1.34 nmol/L), magnesium was 0.45 nmol/L (normal: 0.8–1.0 nmol/L), and serum PTH was 23.9 ng/L (normal: 15–65 ng/L). She had a reduced urine magnesium reabsorption, poor PTH response, and resultant hypocalcemia. In view of that the child suffered from convulsions due to excessive loss of serum magnesium and calcium, she improved after receiving calcium and magnesium supplementation. However, the possibility of genetic metabolic diseases had not been considered. During her follow-up period of 12 years, muscle spasms occurred after infection and diarrhea every year. In addition, the serum magnesium level varied due to the severity of her emesis in the last hospitalization. Her systemic evaluation included normal weight and height, with no signs of deformity. There were no abnormalities in the blood count or results of the thyroid, kidney, and liver function tests. Her serum total calcium was 1.39 nmol/L, ion calcium was 0.42 nmol/L, magnesium was 0.51 nmol/L, and PTH was 15.9 ng/L. During her most recent hospitalization, she was given intravenous Mg^2+^ sulfate and calcium gluconate, and oral magnesium and calcium were provided after discharge. While receiving regular oral treatment, her serum magnesium was >0.5 nmol/L, and her total calcium level was in the normal range. After comprehensive consideration of the clinical and laboratory findings, we thought that the proband may have an inherited metabolic disease. The whole-exome sequencing analysis was suggested.

### Whole-Exome Sequencing

WES was performed by Mygenostics Medical Laboratory Co. Ltd. (Beijing, China) using the peripheral blood samples of the proband and her family. The specific steps were carried out on an Illumina HiSeq 2000 (Bio-Rad, Hercules, CA, USA) by 2 × 100-bp paired-end reads. It was necessary to exclude variants with allele frequencies >1%. The minor allele frequency (MAF) was a comment with the Genome Aggregation Database (gnomAD), dbSNP, 1000 Genomes MAF (Chinese), ExAC, and an in-house MAF database. All candidate variants were clarified in accordance with the American College of Medical Genetics and Genomics (ACMG) criteria (7) and further validated by Sanger sequencing.

### Copy Number Variation Analysis

Using the primer pair sequences listed in Supplementary Table 1, we performed quantitative PCR (qPCR) based methods for expression analysis of the target gene. The experiment was conducted using AceQ qPCR SYBR Green Mix (Vazyme Biotech Co. Ltd) in 7500 Real-Time PCR System (Applied Biosystems, CA, USA). Further, the relative expression of the *TRPM6* gene was normalized to internal control (GAPDH) gene by subtracting the Ct values of E5, E6, and E7 exons. The fold difference in *TRPM6* expression level was calculated using the 2^−Δ*ΔCt*^ method.

### Sanger Sequencing of the c.1638+7T>C Variant

A genomic DNA sample was collected from peripheral blood using the DNA isolation kit (Tiangen, China) according to the standard methods. Primers for PCR amplification of the coding exon 14 and ~200 bp intron boundaries of the *TRPM6* gene were edited by the Primer 5.0 software (F: GCAGATTGTATCAATGGCTGGTCA; R: CTTCTGATCCTTACGCATCCCA). The PCR products were sequenced by BigDye Terminator (Applied Biosystems, CA, USA). In addition, samples from 200 unrelated healthy controls were screened to exclude the non-disease-associated variants in the Chinese population.

### Plasmid Construction

In order to generate a minigene mixture, a pSPL3 minigene reporter vector including a conventional expression system with SD6 and SA2 was used as the resultant mRNA transcripts per previous research, as described in our recent study (8, 9). Briefly, we generated fragments containing the target exons 14 where the splicing variant was located, and 150 bp of flanking intronic regions with XhoI and BamHI restriction sites. Appropriate primers sequences were as follows: Forward: 5′-accagaattctggagctcgagGCACGCATAGATGGAAGCACA-3′; Reverse: 5′-atcaccagatatctgggatccCCCTCTAACCTGCCTCATC ACA-3′. Finally, the fragments were cloned into a pSPL3 vector with the XhoI and BamHI by the ClonExpressTM II One Step Cloning Kit (Vazyme Biotech Co., Ltd). All constructs were confirmed by bidirectional sequencing.

### *In vitro* Splicing Assay

The recombinant constructs (pSPL3-E14-WT and pSPL3-E14-MUT) were transiently transfected in HEK293T, Hela, HepG2, and A549 cells (all obtained from ATCC), using Lip2000 (Invitrogen). Total RNA was extracted from cells using Trizol Reagent (Takara, Japan) 24 h after transient transfection. The first strain cDNA strand was reversely transcribed using the HiScript III transcriptase (Vazyme Biotech Co. Ltd) per the manufacturer's instructions. cDNA was amplified by 2xTaq Master Mix (Vazyme Biotech Co. Ltd) with primers located in the two cassette exons of the pSPL3 vector. The primers sequences used for the experiment were forward SD: 5′-TCTGAGTCACCTGGACAACC-3′ and reverse SA: 5′-ATCTCAGTGGTATTTGTGAGC-3′. The PCR products were visualized on agarose gel and confirmed by sequencing the PCR products after gel extraction.

### Statistical Analysis

All data in this study are shown as means ± SEM. Statistical analysis was made by ANOVA among groups followed by a Bonferroni's comparison test. *p* < 0.05 were considered significant.

## Results

### Genetic Analysis

**Figure 1:**
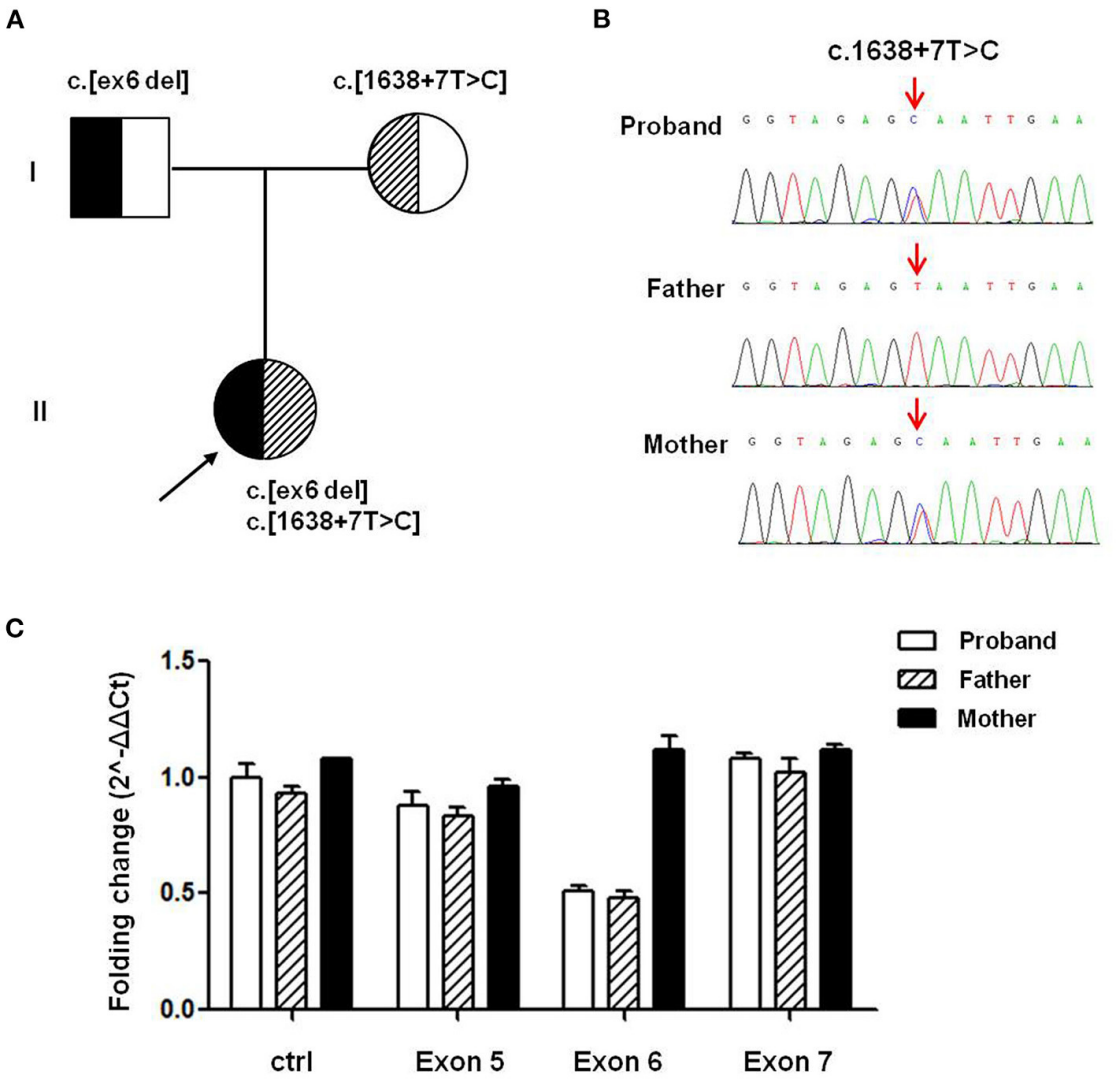
*TRPM6* gene variants identified in a patient with HSH. **(A)** Pedigree of the family from China with two different *TRPM6* variants. **(B)** Direct sequencing shows a splicing variant c.1638+7T>C in intron14 of the *TRPM6* gene (arrows), while the wild-type sequence is also shown (bottom). **(C)** *TRPM6* gene qPCR analysis showed that the proband and her father carried heterozygous exons 6 deletions.

To verify the potential molecular diagnosis, by whole-exome sequencing and subsequent direct sequencing of the *TRPM6* gene, we identified the proband-carried compound heterozygous variants, one is c.545_669 del from her father, another one is c.1638+7T>C inherited from her mother (Figure 1). According to the ACMG criteria, the c.1638+7T>C and c.545_669 del variants were classified as VUS (PM2) and Likely Pathogenic (PM2+PVS1). The c.545_669 del variant caused exon 6 deletion (Ex6 del). The c.1638+7T>C variant was located at the +7 position of the donor site resulting in an abnormal splice of exon 14 by four silico prediction (MaxEntScan, SpliceAI, NNSplice, BDGP). *In silico* analysis indicated that the deletion of exon6 in the *TRPM6* gene leads to a premature stop codon at 191 amino acids (p.(R191^*^)), and this transcript is subject to degradation by the Nonsense-mediated RNA decay (NMD) (https://nmdpredictions.shinyapps.io/shiny/). Copy number variant analysis by qPCR confirmed the large heterozygous loss of *TRPM6* exon 6 in the proband and her father (Figure 1C). The two novel variants have not been reported in the public genomic databases.

### Splicing Minigene Reporter Assay

**Figure 2:**
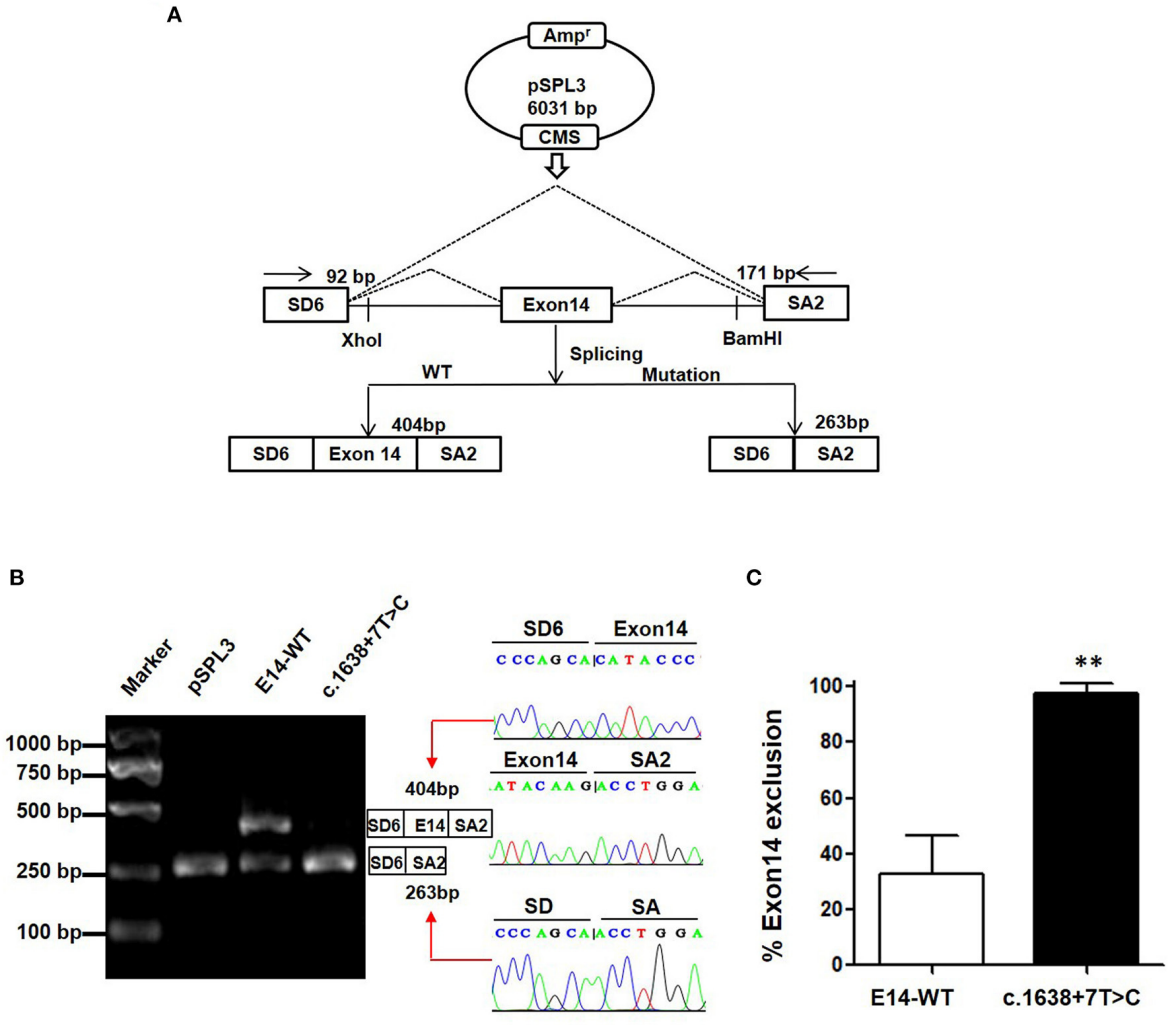
The pSPL3 minigene assay *in vitro* for the splicing variant c.1638+7T>C. **(A)** PCR fragments were, respectively, cloned into the XhoI and BamHI sites of the pSPL3 vector (9). The vector mainly produces two transcripts, wildtype composed of exon SD6, an inserted exon14 and exon SA2 (404 bp), and mutant composed only of exon SD6 and SA2 (263 bp). **(B)** Electrophoresis and sequencing of RT-PCR products of minigene transcripts in HEK293 cells. Lane 1: marker; Lane 2: pSPL3 (263 bp); Lane 3: E14-WT (404 and 263 bp); Lane 4: c.1638+7T>C (404 bp and 263 bp). The cDNA was directly sequenced and indicated that the lower fragment lacked a sequence corresponding to exon 14. **(C)** Quantification of the splicing percentage in HEK293 was calculated on a molar basis as the percentage of exclusion (%) = (lower band/[lower band + upper band]) × 100. Error bars showed SEM (*N* = 3). **p < 0.01.

Minigene assay is an alternative strategy to assess the impact of the splicing variant. The pSPL3 reporter containing a conventional expression system with two cassette exons is a powerful tool to help investigate splicing regulation (Figure 2A). In this study, we designed a pSPL3 minigene assay to clarify the pathogenicity of the c.1638+7T>C variant. After transfecting HEK293T with the minigene plasmids inserting c.1638+7T>C fragment, the total RNA was extracted and then transcribed to cDNA. Subsequently, agarose gel electrophoresis was performed using RT-PCR products. We found that the E14-WT yielded two transcripts and c.1638+7T>C yielded one. The product sequencing revealed that there were 404 bp (SD6+E14+SA2) and 263 bp (SD6+SA2) amplicons, and the difference in size is whether the fragment contained exon 14. This suggests that the c.1638+7T>C is a splicing variant that potentially causes the skipping of exon 14 (SD6+SA2) (Figure 2B). As shown in Figure 2C, the amount of exon 14-skipping transcripts of c.1638+7T>C was significantly increased compared to that of E14-WT (32.8 vs. 97.5%, respectively). To validate whether the transcriptional alteration also occurs in other types of cells, we evaluated the effect of the c.1638+7T>C variant in different tissue (Hela as cervical cells, A549 as pneumonocytes, and HepG2 as hepatocytes). The proportional changes of the exon exclusion in these three cell lines were similar to that in HEK293T (Supplementary Figure 1A). The percentage of exon 14 exclusion of c.1638+7T>C was significantly increased compared with E14-WT (72.9 vs. 98.6% in Hela cells, 5.8 vs. 96.3% in A549 cells, 80.1 vs. 97.4% in HepG2 cells, respectively) (Supplementary Figure 1B). Eventually, our minigene splice assay verified that the non-classic variant of c.1638+7T>C disturbed the normal splicing of *TRPM6* mRNA, which was a pathogenic effect.

## Discussion

In this study, we identified compound heterozygous variants in the *TRPM6* gene [c.1638+7T>C and c.545_669 del (Ex6 del)] as the cause of HSH in a young female patient from China, including hypomagnesemia and secondary hypocalcemia associated with neurological symptoms such as restlessness, tremors, muscle spasms, tetany, and generalized convulsion.

HSH is a rare autosomal recessive disease that leads to electrolyte abnormalities soon after birth (10). More than 50 *TRPM6* mutations contributing to HSH have been reported (11), and only several sporadic cases have been reported in HSH in China (5, 12). *TRPM6* gene comprises 39 exons and encodes a channel protein containing 2,022 amino acids in humans (13). The c.545_669 del (Ex6 del) variant caused exon6 deletion in the *TRPM6* gene leads to a premature stop codon at 191 amino acids (p.(R191^*^)) and is subject to degradation by nonsense-mediated RNA decay (NMD) by prediction, which could be causes degradation of *TRPM6* mRNA transcripts.

In previous studies, *TRPM6* may share structural homology with other TRP channels (14). It is composed of six transmembrane segments, N-terminal and C-terminal domains (15). Our minigene assay indicated that the c.1638+7T>C variant from this subject and her mother caused exon 14 skippings, which would produce the loss of 47 amino acids (codons 1497-1638) and eventually cause a truncated version of the *TRPM6* gene (16). Thus, we upgraded this variant (PM2+PS3) and the c.1638+7T>C variant to likely pathogenic variants, accounting for the proband's HSH. A recent study demonstrated that altering the splice donor site in intron 8 causes N-terminal truncation, which may cause disruption upstream of the TRP domain (17). Note that the transfection of the *TRPM6* mutant found in patients with HSH yielded no detectable currents compared with non-transfected controls, which indicates that the mutant protein has no ion channel function (18).

The primary presentation of HSH was initially reported to manifest as generalized seizures (96%) (19). At the time, serum magnesium was measured in the majority of cases. However, hypomagnesemia was not always considered the primary cause of clinical symptoms or was misinterpreted as a transient symptom. Thus, patients with HSH were administered intravenous calcium for general treatment, which unfortunately resulted in the recurrence of clinical symptoms later in life. Since traditional convulsive medications are ineffective for HSH convulsions, convulsions may be fatal or lead to chronic irreversible neurological complications if not diagnosed early or if the appropriate drug treatment is not performed immediately (20). The proband observed in our clinic predominantly showed seizures and had similar laboratory values for serum magnesium and calcium as noted in previous studies (21). This is an infrequent clinical report of HSH, for this proband had not received regular treatment for ~12 years. Although her blood magnesium content did not reach a healthy level after taking oral magnesium and calcium salt daily, her convulsions were promptly controlled and her life returned to normal. The limitation of this study is that we did not diagnose the disease at an early age, and the follow-up period was quite short. Further understanding of the molecular basis of genetic magnesium homeostasis disorders may provide new insight and approaches to HSH diagnosis and therapy.

## Conclusion

We supported that the compound heterozygous variants in TRPM6 are responsible for HSH in a girl from China. A non-classical splice variant c.1638+7T>C in the intron 14 disturbs the normal TRPM6 mRNA splicing, suggesting that the non-classical splice variant plays a critical role in HSH. This variant is essential for future effective genetic diagnosis. Accurate diagnosis of HSH and appropriate treatment are crucial to preventing irreversible neurological outcomes.

## Data Availability Statement

The datasets for this article are not publicly available due to concerns regarding participant/patient anonymity. Requests to access the datasets should be directed to the corresponding author.

## Ethics Statement

Written informed consent was obtained from the minor(s)' legal guardian/next of kin for the publication of any potentially identifiable images or data included in this article.

## Author Contributions

JS and JZ performed most of *in vitro* experiments and wrote the manuscript with the help of JG. JL and BZ collected the clinical data and performed the NGS analysis. AZ and WG conceived and designed the study. CW and JG contributed to the manuscript drafting and revision in agreement with all other authors. All authors contributed to the article and approved the submitted version.

## Funding

This work was supported by Grants from the National Natural Science Foundation of China (Grant No. 82100719) and the Natural Science Foundation of Jiangsu Province (Grant No. BK20210982).

## Conflict of Interest

The authors declare that the research was conducted in the absence of any commercial or financial relationships that could be construed as a potential conflict of interest.

## Publisher's Note

All claims expressed in this article are solely those of the authors and do not necessarily represent those of their affiliated organizations, or those of the publisher, the editors and the reviewers. Any product that may be evaluated in this article, or claim that may be made by its manufacturer, is not guaranteed or endorsed by the publisher.
