# Supplementary material for: Case Report: A Novel Non-Canonical Splice Site Variant (c.1638+7T>C) in TRPM6 Cause Primary Homagnesemia With Secondary Hocalcemia
Source: Front Pediatr. 2022 May 25;10:834241. doi: 10.3389/fped.2022.834241 (PMC9174589; doi:10.3389/fped.2022.834241)
Supplement: Supplementary file 1 [file Data_Sheet_1.docx]

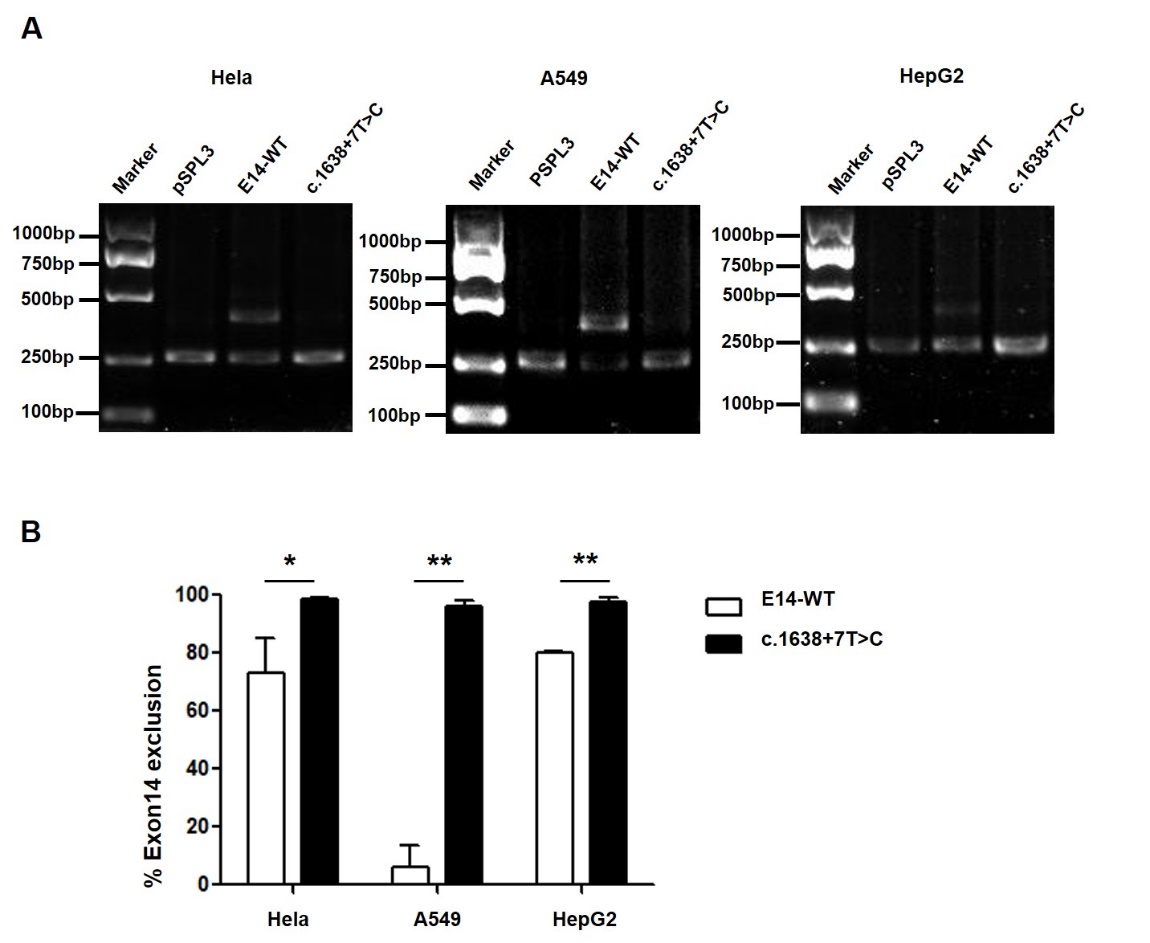


**Supplementary FIGURE 1.** Effect of TRPM6 gene c.1638+7A>G variant by minigene assay. **(A)** Gel electrophoresis of the RT-PCR product of minigene transcripts in Hela, A549 and HepG2 cell. **(B)** Quantification of the splicing percentage in Hela, A549 and HepG2 cell were calculated on a molar basis as the percentage of exclusion (%) = (lower band/[lower band + upper band]) x 100. Error bars represent SEM (n=3). *P < 0.05, **P < 0.01.


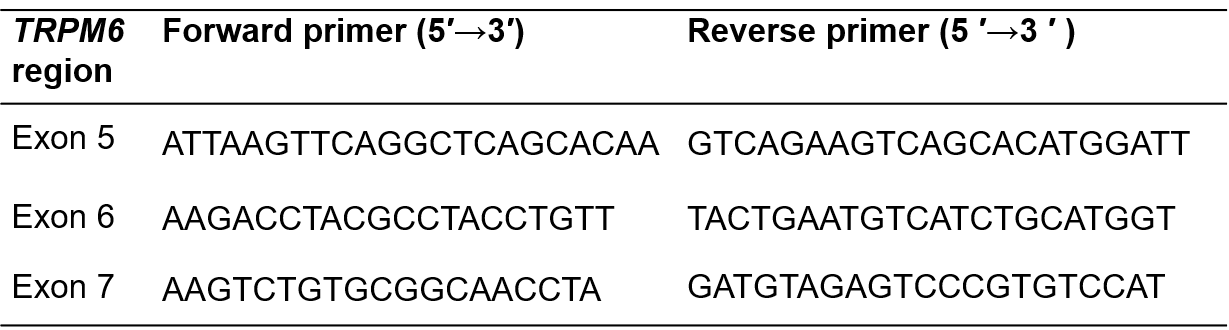
**Supplementary Table 1. Primers used for amplification and sequencing of the coding regions of the TRPM6 gene.**
